# Loss of Household Protection from Use of Insecticide-Treated Nets against Pyrethroid-Resistant Mosquitoes, Benin

**DOI:** 10.3201/eid1807.120218

**Published:** 2012-07

**Authors:** Alex Asidi, Raphael N’Guessan, Martin Akogbeto, Chris Curtis, Mark Rowland

**Affiliations:** London School of Hygiene & Tropical Medicine, London, UK (A. Asidi, R. N’Guessan, C. Curtis, M. Rowland); and; Centre de Recherche Entomologique de Cotonou, Cotonou, Benin (A. Asidi, R. N’Guessan, M. Akogbeto)

**Keywords:** Anopheles gambiae, pyrethroid, insecticide resistance, insecticide treated bed nets, Benin, malaria, mosquitoes, parasites

## Abstract

Pyrethroid resistance is becoming widespread in *Anopheles gambiae* mosquitoes, coinciding with expanded use of insecticide-treated nets (ITNs) throughout Africa. To investigate whether nets in use are still protective, we conducted household trials in northern and southern Benin, where *An. gambiae* mosquitoes are susceptible and resistant, respectively, to pyrethroids. Rooms were fitted with window traps and monitored for mosquito biting and survival rates before and after the nets were treated with pyrethroid. Sleeping under an ITN in the location with resistant mosquitoes was no more protective than sleeping under an untreated net, regardless of its physical condition. By contrast, sleeping under an ITN in the location with susceptible mosquitoes decreased the odds of biting by 66%. ITNs provide little or no protection once the mosquitoes become resistant and the netting acquires holes. Resistance seriously threatens malaria control strategies based on ITN.

## Abstract 2

Restoring protection requires innovation combining pyrethroids and novel insecticides.

Insecticide-treated nets (ITNs) and long-lasting insecticidal nets (LLINs) are the primary interventions for preventing malaria in sub-Saharan Africa (*1*,*2*). Nets accumulate holes through wear and tear during the course of everyday use, but the pyrethroid treatment continues to provide personal protection and to reduce vector capacity through excito-repellency and the killing of mosquitoes that contact the net (*3*,*4*). During the last decade, pyrethroid resistance in *Anopheles gambiae* mosquitoes became widespread in western Africa and spread to or developed in eastern Africa (*5*–*9*). As coverage of LLINs expands across the continent under programs supported by the President’s Malaria Initiative and Global Fund (*10*), resistance will inevitably increase (*11*–*13*).

Although resistance is perceived as a serious threat to the future of malaria control, the current distribution of resistance is patchy, and its severity seems to differ from 1 location to another. In the western African country of Benin, pyrethroid resistance has evolved in the M (Mopti) molecular form of *An. gambiae* mosquitoes that appears to combine the knockdown resistance (*kdr*) gene with oxidase mechanisms (*14*,*15*). Carriers of this resistance were not controlled by pyrethroid treatments in experimental hut trials of ITNs or the leading brands of LLINs, PermaNet 2.0 (Vestergaard Frandsen SA, Aarhus, Denmark) and Olyset (Sumitomo Chemicals, Osaka, Japan) (*16*,*17*). However, further west in Côte d’Ivoire, the *kdr* in *An. gambiae* S (Savannah) form mosquitoes conferred only limited resistance, and trials of ITNs continued to protect against mosquito blood feeding (biting) and malaria transmission by this species (*18*–*20*).

Results from experimental hut trials in Benin raise an alarm. Of key concern is whether ITNs that are subject to wear and tear under everyday household conditions fail to protect ITN users now that *An. gambiae* mosquitoes are becoming resistant. Modern mosquito nets lack physical durability, and household nets can accrue an average of 12–20 holes during 1–2 years of use (*21*). Net replacement schemes struggle to meet demand at this level of deterioration and attrition. To assess protection conferred by in-use polyester nets, we compared nets in households of northern Benin, where *An. gambiae* mosquitoes are mostly susceptible to pyrethroids, with nets in households of southern Benin, where *An. gambiae* mosquitoes are mostly resistant (*7**,**16*,*22*). Residents of the selected households were all regular users of nets.

## Materials and Methods

### Study Sites

Three suburbs (Ladji, Fifadji, and Abomey Calavi) of Cotonou in southern Benin support breeding of mosquitoes of *An. gambiae* M form that is mostly pyrethroid resistant with a high frequency of *kdr* (>90%) (*14*,*22*). Malanville, 800 km north of Cotonou, is situated in an area in which mosquitoes are mainly pyrethroid susceptible, where *An. gambiae* M form mosquitoes show a *kdr* frequency of <0.05 (*7**,**14*).

### Selection of Households and Torn Nets

We selected 3–5 households from each site. The criteria for selection were that each house contain a sleeping room with a close-fitting door and a window suitable for fitting a mosquito exit trap and in which occupants possessed >1 worn nets under regular use. The points of entry for mosquitoes were through open doors or eave gaps between walls and roofs. Nets were made of polyester, cotton, or nylon and contained holes of various sizes and number. Before inclusion, the nets were subjected to World Health Organization cone bioassays by using a laboratory-susceptible strain of *An. gambiae* to detect pyrethroid residue. Only untreated nets or nets that had lost their insecticide through washing were retained for the study.

Household members gave informed consent to participate in the study and were provided with chemoprophylaxis throughout. The London School of Hygiene & Tropical Medicine and the Benin national ethics committees granted ethics approval.

### Mosquito Exit Window Traps

Unidirectional window traps were fixed to window frames for collecting exiting mosquitoes. Each trap consisted of a 30-cm–sided metal frame covered with polyester netting, with 1 side drawn into a funnel to direct mosquitoes into the trap (*23*). The trap was fixed to a plywood sheet that could be fitted to window frames of differing sizes. The traps were placed before dusk and emptied of mosquitoes at 7 am.

### Treatment of Mosquito Nets

Nets were treated with a microencapsulated formulation of lambdacyhalothrin (Icon 10 CS, Syngenta, Basel, Switzerland). The standard rate of 18 mg/m^2^ was used.

### Mosquito Collection

We conducted the trials during May and June 2008 at the southern sites and during July and August 2008 at the northern site. Rooms of selected houses containing untreated nets were fitted with traps and monitored for 5 consecutive nights to assess baseline mosquito density and blood-feeding and death rates. Nets were then treated with lambdacyhalothrin and monitored for 5 additional nights. Houses that attracted too few mosquitoes during baseline monitoring were dropped. Each morning, mosquitoes were collected from the window traps by mouth aspirator and transferred to paper cups and provided with sugar solution. Indoor resting mosquitoes were then collected from white floor sheets after the windows were sealed off and the rooms were sprayed with a nonresidual pyrethroid. Mosquitoes were identified to species and recorded as blood fed or unfed by microscopy. Scoring of blood-feeding rates was pooled for window trap and room collections. Death rates of the exit trap collections were determined after a 24-hour holding period. *An. gambiae* mosquitoes were identified to species and molecular form by using the method of Favia et al. (*24*) and genotyped for *kdr* by using the method of Martinez-Torres et al. (*25*).

### Data Analysis

We assessed the effect of pyrethroid-treated nets on the proportions of *An. gambiae* blood-feeding or killed mosquitoes using a random effects generalized linear mixed model, recording the proportions of female mosquitoes before treatment as the baseline (control) group and the proportions after treatment as the test group. The model comprised 4 independent variables: treatment, number of holes per net, total area of all holes in the net under test, and number of persons in the household. Random effects in the model also accounted for repeated sampling over several days and the number of persons sleeping in the room. Regional differences in the condition of nets and in household size between the sites with resistant and susceptible mosquitoes were analyzed by using the Wilcoxon rank sum test. All statistical analyses were conducted by using STATA 9 software (STATA Corp., College Station, TX, USA).

## Results

### Baseline Characteristics of Mosquito Nets and Sleepers

Eleven households at the southern sites (where mosquitoes are resistant) and 5 households at the northern site (where mosquitoes are susceptible) participated in the study. Each household contributed 1 sleeping room and 1 net to the study. Numbers of holes per net recorded at the southern and northern sites did not differ (p = 0.41) (Table 1). The area of holes per net was significantly smaller for nets from the south (p = 0.0013) (Table 2). Household size in the south was twice that in the north (p = 0.025).

**Table 1:** Baseline characteristics showing condition of selected mosquito nets in households in northern sites, where mosquitoes are pyrethroid susceptible, vs. southern sites, where mosquitoes are pyrethroid resistant, Benin, 2008*

Variable	Northern site	Southern sites	Difference (95% CI)	p value
Households, no.	5	11	NA	NA
Household members, average no. (range)	2.2 (1–3)	5.1 (2–7)	2.9 (1.4–4.4)	0.025
Holes in nets				
Average no. (range)	10.2 (5–13)	9.5 (5–25)	0.65 (–5.3 to 6.6)	0.41
Average size, cm^2^ (range)	28 (11–49)	11 (5–20)	15 (7–21)	0.0013

*NA, not applicable.

**Table 2:** Protection against *Anopheles gambiae* s.l. mosquitoes for persons sleeping under in-use mosquito nets before and after treatment with 18 mg/m^2^ lambdacyalothrin in houses in northern vs. southern sites, Benin, 2008*

Area, net condition	Treatment of nets	Blood fed, no. (%)	OR (95% CI)	p value	aOR (95% CI)	p value
Northern (pyrethroid-susceptible mosquitoes)	Before	810 (46)	1		1	
	After	1,041 (16)	0.22 (0.18–0.28)	<0.001	0.34 (0.26–0.44)	<0.001
No. holes						
<10	Before	503 (45)	1		1	
	After	850 (14)	0.20 (0.16–0.27)	<0.001	0.26 (0.20–0.34)	<0.001
>10	Before	307 (48)	1		1	
	After	191 (24)	0.34 (0.23–0.51)	<0.001	0.37 (0.27–0.64)	<0.001
Size of holes, cm^2^						
<15	Before	59 (36)	1		1	
	After	217 (17)	0.38 (0.20–0.73)	0.003	0.38 (0.20–0.73)	0.003
>15	Before	751 (47)	1		1	
	After	824 (16)	0.21 (0.17–0.27)	<0.001	0.21 (0.17–0.27)	<0.001
Southern (pyrethroid-resistant mosquitoes)	Before	268 (20)	1		1	
	After	424 (23)	1.19 (0.81–1.73)	0.37	1.14 (0.73–1.76)	0.57
No. holes						
<10	Before	111 (18)	1		1	
	After	200 (21)	1.15 (0.55–1.67)	0.28	1.17 (0.62–1.81)	0.31
>10	Before	165 (27)	1		1	
	After	224 (25)	0.89 (0.56–1.42)	0.64	0.89 (0.56–1.41)	0.63
Size of holes, cm^2^						
<15	Before	115 (19)	1		1	
	After	189 (23)	1.31 (0.74–2.36)	0.35	2.59 (1.26–5.37)	0.01
>15	Before	153 (21)	1		1	
	After	235 (23)	1.10 (0.67–1.8)	0.70	1.09 (0.18–1.80)	0.713

*OR, odds ratio; aOR, OR adjusted for condition of nets and household size.

### Efficacy of Mosquito Nets Before and After Treatments

During the 2-month trial, 692 *An. gambiae* mosquitoes; 2,271 *Culex quinquefasciatus* mosquitoes; and small numbers of *Mansonia uniformis*, *An. pharoensis*, and *Aedes aegypti* mosquitoes were collected at the southern sites. At the northern site, 1,856 *An. gambiae* mosquitoes, 1,051 *Mansonia* spp. mosquitoes, and small numbers of *An. funestus* and *Ae. aegypti* mosquitoes were collected. Only the malaria vector *An. gambiae* was analyzed further.

The blood-feeding rate of *An. gambiae* mosquitoes under untreated nets was higher in the north (46%) than in the south (20%) (Table 2), probably because of the larger size of holes in nets in the north. At the northern site (susceptible mosquitoes), the odds of blood feeding were lower after treatment than before treatment with or without adjustment for other covariates (adjusted odds ratio 0.34; 95% CI 0.26–0.44; p<0.001) (Table 2). The overall protective effect of treatment was 66% (95% CI 56%–74%). The OR for nets with smaller and larger areas of holes indicated that ITNs provided similar levels of protection against the susceptible mosquito population regardless of the condition of the nets (Table 2).

At the southern sites, where mosquitoes are resistant, we found no evidence that sleeping under a treated net was more protective than sleeping under an untreated net (adjusted odds ratio 1.14; 95% CI 0.73–1.76; p = 0.566) (Table 2). There was no difference in blood feeding rates between nets that had a higher number and nets that had a lower number of holes. Nor was there any difference between nets that had a higher surface area or lower surface area of holes. These findings indicated that regardless of physical condition, treated nets provided no additional protection over that of untreated nets.

Mosquito mortality rates in the exit traps at the northern site (susceptible mosquitoes) were 8% before insecticide treatment of the nets and 70% after treatment. Mosquito mortality rates at the sites where they are pyrethroid resistant were similar before and after treatment of the nets and did not exceed 12% (Figure).

**Figure:**
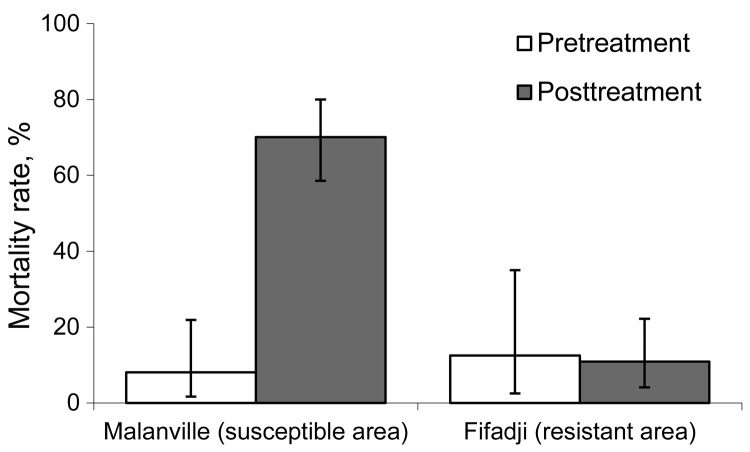
Death rates of *Anopheles gambiae* s.l. mosquitoes collected in exit traps at Mallanville (where mosquitoes are pyrethroid susceptible) in northern Benin and Fifadji (where mosquitoes are pyrethroid resistant) in southern Benin, 2008. Error bars indicate 95% CIs.

### Species, Molecular, and *kdr* Genotyping

PCR identified *An. gambiae* s.s. as the main sibling species at southern and northern sites (Table 3). *An. arabiensis* mosquitoes were present as a minor sibling species at the northern site. The M form of *An. gambiae* mosquitoes predominated at all sites (83%). The frequency of *kdr* was 0.80 in the south and 0.10 in the north.

**Table 3:** Characteristics of *Anopheles gambiae* s.l. mosquitoes collected from study households in southern and northern Benin, 2008*

Location, mosquito resistance	Species			Molecular form of *An. gambiae*			*kdr* genotype			*kdr* frequency
	*An. arabiensis*	*An. gambiae*		M	S		SS	RS	RR	
Northern, susceptible	5	30		27	3		28	5	1	0.10
Southern, resistance	0	60		56	4		10	3	45	0.80

*Values are no. mosquitoes except as indicated.

## Discussion

In this comparative study in areas of contrasting pyrethroid resistance and susceptibility, we used vector blood feeding as a surrogate for malaria risk and demonstrated that ITNs lose their capacity to provide protection once *An. gambiae* M form develops pyrethroid resistance of the type found in southern Benin (*16*). These findings clearly show that ITNs in local use fail to protect against *An. gambiae* populations that contain *kdr* resistance at high frequency. The mechanisms of resistance in southern Benin are complex, and metabolic resistance appears to contribute (*14*–*16*). The demonstration of contrasting blood-feeding and survival rates between resistant and susceptible *An. gambiae* mosquitoes in the household trial corroborates findings and predictions from earlier experimental hut studies in southern and northern Benin and confirms the veracity of experimental huts as a tool for predicting protection or control in the home (*16*,*22*).

We chose to use ordinary household nets rather than new or intact nets. Intact nets might have provided barrier protection against resistant and susceptible mosquitoes, but such a trial would not have reflected local reality. Household nets are inevitably subject to wear and tear, and several studies have documented the association between naturally damaged ITNs and mosquito blood-feeding rates. Before the advent of ITNs, Port and Boreham (*26*), in an experimental hut study of bed nets previously used by local Gambians, found a strong correlation between blood feeding and the number and size of holes. More recently, Irish et al. (*27*), in an experimental hut trial of treated nets against pyrethroid-resistant *Cx. quinquefasciatus* mosquitoes, found an association between the proportion of mosquitoes blood feeding and the number of holes in the ITN. Cross-sectional parasite prevalence surveys in Equatorial Guinea showed that children sleeping under intact ITNs were protected against infection with *Plasmodium falciparum* but that the level of protection progressively decreased as the nets’ condition deteriorated (*28*). Our study also stratified nets according to condition, and the analysis showed that persons sleeping under ITNs with holes in areas with pyrethroid-resistant mosquitoes had the same risk from mosquitoes as did persons using untreated nets, whereas in areas of pyrethroid susceptibility, the ITNs remained protective regardless of physical condition. As nets inevitably acquire holes over time, the loss of the nets’ integrity will be felt most strongly in areas with resistant mosquitoes, and the community will be put at greater risk for malaria.

Campaigns of universal LLIN coverage aim to protect the families least able to afford nets (*29*). With the loss of net integrity over time, malaria transmission will continue across all age groups. Our results predict that mass distribution campaigns of LLINs would benefit populations in areas of pyrethroid susceptibility but are unlikely to control malaria in areas of high resistance. In villages of rural Benin, where pyrethroid resistance in *An. gambiae* mosquitoes is moderate (*kdr* frequency averaging 40%), the regular use of LLINs has had some effect on the prevalence of malaria among children <5 years of age (*30*). We anticipate that in villages with *kdr* frequency >80% that are subject to high rates of malaria transmission, as in the southern provinces (*22*,*31*), the effects on the community of LLINs on malaria would be compromised among families who have poor-quality ITNs.

Sustained protection by any LLIN depends on 2 factors: the rate of loss of insecticide residue from the fibers and the retention of textile integrity. Our research shows that the emphasis placed by the World Health Organization and net manufacturers on developing nets that retain insecticide after recurrent washing is overlooking the role of net durability on effectiveness. A net that retains insecticide after multiple washes or 3 years of use is of no benefit if, before this period, the physical condition of the net and the holes that accumulate mean that in locations with high levels of resistance the net has lost the capacity to protect. During household use, polyester- and polyethylene-based LLINs acquiring holes within the first year and are starting to be discarded after 2 years (*21*,*28*,*32*). LLIN manufacturers need to create new types of fiber or increase the tensile strength to give better resilience against tearing or acquiring holes. Any such product should have a strong commercial advantage.

Resistance capable of undermining the effective use of LLINs is not confined to southern Benin. With the growing coverage of LLINs, the continuing selection of resistance in mosquitoes and its spread to mosquitoes in neighboring provinces and countries is inevitable. Restoring protection of LLINs requires innovation that combines pyrethroids and novel insecticides to which this form of *An. gambiae* mosquitoes shows no resistance.

## References

[1] Lengeler C. Insecticide-treated bed nets and curtains for preventing malaria. Cochrane Database Syst Rev. 2004;CD000363. 15106149 10.1002/14651858.CD000363.pub2

[2] Hill J, Lines J, Rowland M. Insecticide-treated nets. Adv Parasitol. 2006;61:77–128. 10.1016/S0065-308X(05)61003-2 16735163

[3] Lines JD, Myamba J, Curtis CF. Experimental hut trials of permethrin-impregnated mosquito nets and eave curtains against malaria vectors in Tanzania. Med Vet Entomol. 1987;1:37–51. 10.1111/j.1365-2915.1987.tb00321.x 2979519

[4] Magesa SM, Wilkes TJ, Mnzava AE, Njunwa KJ, Myamba J, Kivuyo MD, Trial of pyrethroid impregnated bednets in an area of Tanzania holoendemic for malaria. Part 2. Effects on the malaria vector population. Acta Trop. 1991;49:97–108. 10.1016/0001-706X(91)90057-Q 1680284

[5] Chandre F, Manguin S, Brengues C, Dossou Yovo J, Darriet F, Diabate A, Current distribution of a pyrethroid resistance gene (*kdr*) in *Anopheles gambiae* complex from west Africa and further evidence for reproductive isolation of the Mopti form. Parassitologia. 1999;41:319–22. 10697876

[6] Sharp BL, Ridl FC, Govender D, Kuklinski J, Kleinschmidt I. Malaria vector control by indoor residual insecticide spraying on the tropical island of Bioko, Equatorial Guinea. Malar J. 2007;6:52. 10.1186/1475-2875-6-52 17474975 PMC1868751

[7] Djogbénou L, Pasteur N, Akogbéto M, Weill M, Chandre F. Insecticide resistance in the *Anopheles gambiae* complex in Benin: a nationwide survey. Med Vet Entomol. 2011;25:256–67. 10.1111/j.1365-2915.2010.00925.x 21155858

[8] Mathias DK, Ochomo E, Atieli F, Ombok M, Bayoh MN, Olang G, Spatial and temporal variation in the kdr allele L1014S in *Anopheles gambiae* s.s. and phenotypic variability in susceptibility to insecticides in western Kenya. Malar J. 2011;10:10. 10.1186/1475-2875-10-10 21235783 PMC3029224

[9] Ranson H, N’Guessan R, Lines J, Moiroux N, Nkuni Z, Corbel V. Pyrethroid resistance in African anopheline mosquitoes: what are the implications for malaria control? Trends Parasitol. 2011;27:91–8. 10.1016/j.pt.2010.08.004 20843745

[10] World Health Organization. World malaria report 2011. Geneva: The Organization; 2011.

[11] Czeher C, Labbo R, Arzika I, Duchemin JB. Evidence of increasing Leu-Phe knockdown resistance mutation in *Anopheles gambiae* from Niger following a nationwide long-lasting insecticide-treated nets implementation. Malar J. 2008;7:189. 10.1186/1475-2875-7-189 18817574 PMC2562389

[12] Ramphul U, Boase T, Bass C, Okedi LM, Donnelly MJ, Müller P. Insecticide resistance and its association with target-site mutations in natural populations of *Anopheles gambiae* from eastern Uganda. Trans R Soc Trop Med Hyg. 2009;103:1121–6. 10.1016/j.trstmh.2009.02.014 19303125

[13] Protopopoff N, Verhaeghen K, Van Bortel W, Roelants P, Marcotty T, Baza D, A significant increase in *kdr* in *Anopheles gambiae* is associated with an intensive vector control intervention in Burundi highlands. Trop Med Int Health. 2008;13:1479–87. 10.1111/j.1365-3156.2008.02164.x 18983277

[14] Corbel V, N’Guessan R, Brengues C, Chandre F, Djogbenou L, Martin T, Multiple insecticide resistance mechanisms in *Anopheles gambiae* and *Culex quinquefasciatus* from Benin, west Africa. Acta Trop. 2007;101:207–16. 10.1016/j.actatropica.2007.01.005 17359927

[15] Djouaka RF, Bakare AA, Coulibaly ON, Akogbeto MC, Ranson H, Hemingway J, Expression of the cytochrome P450s, CYP6P3 and CYP6M2 are significantly elevated in multiple pyrethroid resistant populations of *Anopheles gambiae* s.s. from southern Benin and Nigeria. BMC Genomics. 2008;9:538. 10.1186/1471-2164-9-538 19014539 PMC2588609

[16] N’Guessan R, Corbel V, Akogbéto M, Rowland M. Reduced efficacy of insecticide-treated nets and indoor residual spraying for malaria control in pyrethroid resistance area, Benin. Emerg Infect Dis. 2007;13:199–206. 10.3201/eid1302.060631 17479880 PMC2725864

[17] N’Guessan R, Asidi A, Boko P, Odjo A, Akogbeto M, Pigeon O, An experimental hut evaluation of PermaNet 3.0, a deltamethrin-piperonyl butoxide combination net, against pyrethroid-resistant *Anopheles gambiae* and *Culex quinquefasciatus* mosquitoes in southern Benin. Trans R Soc Trop Med Hyg. 2010;104:758–65. 10.1016/j.trstmh.2010.08.008 20956008

[18] Asidi AN, N’Guessan R, Hutchinson RA, Traoré-Lamizana M, Carnevale P, Curtis CF. Experimental hut comparisons of nets treated with carbamate or pyrethroid insecticides, washed or unwashed, against pyrethroid-resistant mosquitoes. Med Vet Entomol. 2004;18:134–40. 10.1111/j.0269-283X.2004.00485.x 15189238

[19] Asidi AN, N’Guessan R, Koffi AA, Curtis CF, Hougard JM, Chandre F, Experimental hut evaluation of bednets treated with an organophosphate (chlorpyrifos-methyl) or a pyrethroid (lambdacyhalothrin) alone and in combination against insecticide-resistant *Anopheles gambiae* and *Culex quinquefasciatus* mosquitoes. Malar J. 2005;4:25. 10.1186/1475-2875-4-25 15918909 PMC1156935

[20] Henry MC, Assi SB, Rogier C, Dossou-Yovo J, Chandre F, Guillet P, Protective efficacy of lambda-cyhalothrin treated nets in *Anopheles gambiae* pyrethroid resistance areas of Côte d’Ivoire. Am J Trop Med Hyg. 2005;73:859–64. 16282294

[21] World Health Organization. Report of the Twelfth WHOPES Working Group Meeting, 8–11 December 2008. Geneva: The Organization; 2009.

[22] Yadouleton AW, Padonou G, Asidi A, Moiroux N, Biol-Banganna S, Corbel V, Insecticide resistance status in *Anopheles gambiae* in southern Benin. Malar J. 2010;9:83. 10.1186/1475-2875-9-83 20334637 PMC2858214

[23] Bar-Zeev M, Self LS. A note on the use of window traps as a tool for evaluating insecticides. Mosq News. 1966;26:205–7.

[24] Favia G, della Torre A, Bagayoko M, Lanfrancotti A, Sagnon N, Touré YT, Molecular identification of sympatric chromosomal forms of *Anopheles gambiae* and further evidence of their reproductive isolation. Insect Mol Biol. 1997;6:377–83. 10.1046/j.1365-2583.1997.00189.x 9359579

[25] Martinez-Torres D, Chandre F, Williamson MS, Darriet F, Berge JB, Devonshire AL, Molecular characterization of pyrethroid knockdown resistance (*kdr*) in the major malaria vector *Anopheles gambiae* s.s. Insect Mol Biol. 1998;7:179–84. 10.1046/j.1365-2583.1998.72062.x 9535162

[26] Port GR, Boreham PFL. The effect of bednets on feedng by *Anopheles gambiae* Giles (Diptera: Culicidae). Bull Entomol Res. 1982;72:483–8. 10.1017/S0007485300013663

[27] Irish S, N’Guessan R, Boko PM, Metonnou C, Odjo A, Akogbeto M, Loss of protection with insecticide-treated nets against pyrethroid-resistant *Culex quinquefasciatus* mosquitoes once nets become holed: an experimental hut study. Parasit Vectors. 2008;1:17. 10.1186/1756-3305-1-17 18564409 PMC2459145

[28] Rehman AM, Coleman M, Schwabe C, Baltazar G, Matias A, Gomes I, How much does malaria vector control quality matter: the epidemiological impact of holed nets and inadequate indoor residual spraying. PLoS ONE. 2011;6:e19205. 10.1371/journal.pone.0019205 21559436 PMC3084796

[29] Tsuang A, Lines J, Hanson K. Which family members use the best nets? An analysis of the condition of mosquito nets and their distribution within households in Tanzania. Malar J. 2010;9:211. 10.1186/1475-2875-9-211 20663143 PMC2918626

[30] Damien GB, Djènontin A, Rogier C, Corbel V, Bangana SB, Chandre F, Malaria infection and disease in an area with pyrethroid-resistant vectors in southern Benin. Malar J. 2010;9:380. 10.1186/1475-2875-9-380 21194470 PMC3224346

[31] Nahum A, Erhart A, Mayé A, Ahounou D, van Overmeir C, Menten J, Malaria incidence and prevalence among children living in a peri-urban area on the coast of Benin, west Africa: a longitudinal study. Am J Trop Med Hyg. 2010;83:465–73. 10.4269/ajtmh.2010.09-0611 20810805 PMC2929036

[32] Kilian A, Byamukama W, Pigeon O, Atieli F, Duchon S, Phan C. Long-term field performance of a polyester-based long-lasting insecticidal mosquito net in rural Uganda. Malar J. 2008;7:49. 10.1186/1475-2875-7-49 18355408 PMC2330059

